# Smart Grid as a Service: A Discussion on Design Issues

**DOI:** 10.1155/2014/535308

**Published:** 2014-08-27

**Authors:** Hung-Lin Chao, Chen-Chou Tsai, Pao-Ann Hsiung, I-Hsin Chou

**Affiliations:** ^1^Department of Computer Science and Information Engineering, National Chung Cheng University, No. 168 University Road, Min-Hsiung Township, Chiayi County 62102, Taiwan; ^2^The Examination Yuan of ROC, No. 1 Shiyuan Road, Wenshan District, Taipei City 11601, Taiwan

## Abstract

Smart grid allows the integration of distributed renewable energy resources into the conventional electricity distribution power grid such that the goals of reduction in power cost and in environment pollution can be met through an intelligent and efficient matching between power generators and power loads. Currently, this rapidly developing infrastructure is not as “smart” as it should be because of the lack of a flexible, scalable, and adaptive structure. As a solution, this work proposes smart grid as a service (SGaaS), which not only allows a smart grid to be composed out of basic services, but also allows power users to choose between different services based on their own requirements. The two important issues of service-level agreements and composition of services are also addressed in this work. Finally, we give the details of how SGaaS can be implemented using a FIPA-compliant JADE multiagent system.

## 1. Introduction

Electricity power grids are responsible for distributing electrical power to users, via substations. The traditional grid is dumb and static because it does not know how much and when power is required by users. Thus, it has to cope with the worst case of peak power usage, for example, during the noontime of a summer day. However, the peak usage might be much higher than the normal requirement. Thus, the amount of power generated is much higher than the average required, which results in not only a waste of electrical power, but also increases in environment pollution. As a complementary effort, smart grid tries to provide a more intelligent and efficient matching between the power generation with the power demands from loads. Thus, the power generation utility will not need to support peak power demands as the power demands above the normal average amount can be met through distributed energy resources (DER) and energy storage systems (ESS).

Nevertheless, the status quo of smart grid design is still far from being mature because of the lack of a basic infrastructure for communication and adaptation. In this work, we try to leverage on the popular* service-oriented architecture* (SOA) for filling in this gap of smart grid infrastructure. SOA allows greater flexibility in system design and development. It also makes a system design more reliable due to the ease in which functions can be recovered through either resubscription of new services or recovery of failed services.

**Figure 1:**
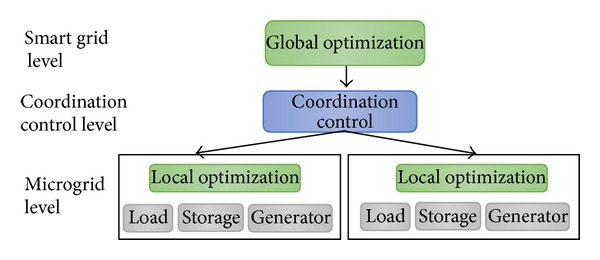
Conventional smart grid architecture.

As shown in Figure 1, a conventional smart grid architecture has the functions all embedded into the system design. For example, global optimization and local optimization are predesigned into the system. There is no flexibility in the way in which optimization can be performed. If the method is genetic algorithm (GA) based, then the method is designed into the system and the microgrids have no other choice but to accept the optimization method designed into the system. However, in a service-oriented design of smart grid architecture, the microgrids can choose whether to use GA-based optimization or bidding or simple matching. Accordingly, we propose a novel smart grid as a service (SGaaS) which leverages the service-oriented architecture of systems. SGaaS allows greater flexibility in system design and development. The two important issues of service-level agreements and composition of services are also addressed in this work.

In the rest of this paper, we first give an overview on the smart grid design and also on how services are being designed for specific functions of the smart grid. These will be covered in Section 2. In Section 3, we discuss how smart grid as a service can be designed and deployed using contemporary design techniques. In Section 4, we discuss two important issues in SOA, namely, service-level agreement and service composition. In Section 5, we give the implementation of how the proposed SOA for smart grid can be realized. In Section 6, we conclude with some future work.

## 2. Previous Work

Service-oriented architecture is becoming the trend in system design due to its many benefits. The most evident proof of this is the cloud computing infrastructure based on the three tiers of IaaS (infrastructure as a service), PaaS (platform as a service), and SaaS (software as a service) [1]. Further cloud computing is becoming the backbone of most applications as they become more and more mobile. For example, the power information from smart meters is currently mostly uploaded to cloud servers, which are then accessed remotely via web services.

As far as the adoption of service-oriented architecture for smart grids, there is still very little work in this area. Verschueren et al. [2] proposed a service architecture for smart end-user devices which are the appliances plugged into distributed energy management systems. The proposed architecture focuses on the integration of end-user devices and the service. However, this approach does not address interface discrepancy issues. Chen et al. [3] proposed an infrastructure for service-oriented advance metering in smart grids. The authors adopted a role-based access control mechanism to guarantee secure access to smart grids. Pham et al. [4] proposed a flexible service-oriented architecture for power system asset management. This work used the Bender's decomposition decision algorithm to decompose highly complex decision processes into smaller ones. However, the above-mentioned approaches do not deal with the issues of end-to-end quality-of-service guarantee violation, which is an important issue in designing service-oriented architecture. Enose [5] proposed a unified management system for smart grids. The author pointed out how a unified management system is an important part in building a smart grid environment and discussed the role in service management layer of proposed approach but does not address issues about information conglomeration.

Table 1 lists the comparison of SGaaS with the mentioned service-oriented architectures. We compared four features, including hierarchical architecture, trading process, system design feature, and simulator. For hierarchical architecture, SGaaS proposes a three-layer architecture, including smart grid level (SGL) for global optimization such as minimizing global pollution or global cost, coordination control level (CCL) for maintaining reliability and security in smart grids, and microgrid level (MGL) for monitoring the state of end-user device. Unlike the other work, the architectures proposed by Chen et al. [3] and Pham et al. [4] do not consider CCL and thus do not address security problems.

The trading process is an important issue in designing SOA of smart grids. However, the above-mentioned works do not address this issue. In SGaaS, three trading mechanisms are supported, namely, auction, matching, and optimization, the selection of which can be made during trading SLA negotiation at the smart grid level. SGaaS provides a flexible framework that can adapt the optimization method for different objectives at each level. As for system design and simulator, Verschueren et al. [2] considers the control-oriented aspect to integrate the services in smart grid. The authors adopted the OMNeT++ simulator to simulate the energy flow of power grid and integrate information and communication technology (ICT) network for controlling information flow. Chen et al. [3] proposed a generic service interfacing method for service standardization for both provider and customer. Pham et al. [4] proposed a decision algorithm for power grid, which makes the highly complex decision processes more efficiently. This work uses the AREVA OTS simulator to simulate the power grid and adopts the MATLAB to implement the decision algorithm. Enose [5] proposed a unified management approach to integrate various services in different layers. In SGaaS, we provide a multiagent system-based service-oriented architecture simulator to simulate the entire smart grid environment.

There is a large body of work focusing on how multiagent systems are used in negotiating the service-level agreements (SLA) [6–10]. Giri et al. [6], Ouelhadj et al. [7], He et al. [8], and Chhetri et al. [9] have all applied multiagent systems to SLA negotiations. Further, SLA negotiations in cloud computing are currently also under investigation [10].

The design of a smart grid [11–14] can be segregated into three layers, including (1) Application Layer that supports the integration of smart grid services and the monitoring of parameters and status, (2) Logic Layer that supports the configuration of smart grids and the management of policies, and (3) Simulation Layer that supports the agent-based simulation of generators and loads such that complex scenarios can be evaluated. In the 2005 World Expo held in Japan, there was a demonstration of how smart grids can be applied to real life [13]. The optimization algorithms used in this demonstration were Tabu search (TS) and genetic algorithm (GA), which when integrated with smart grid simulation can be used to reduce the time required for selecting an optimal power distribution route. The GA algorithm could produce a near optimal solution in a very short time, while the TS algorithm was slower but produced a more optimal solution.

Recently, multiagent systems (MAS) have been applied to the modeling and evaluation of smart grid systems [15–19]. In this approach, generators, loads, and storages are represented by agents, which communicate with each other in a distributed manner, while achieving global goals. MAS-based simulators of smart grids generally conform to the standards set by the Foundation for Intelligent Physical Agents (FIPA). Java Agent Development Framework (JADE) [20] and ZEUS [21] are two FIPA-compliant MAS platforms that have been used to simulate smart grids. MAS-based simulations allow smart grids to achieve global goals such as cost minimization, pollution minimization, and network loss reduction in a distributed manner. Even the hierarchical hybrid control of smart microgrids is modeled and simulated by MAS [17]. Trading features among loads and generators along with auctioning are also modeled using MAS [15, 16, 18]. Load shedding methods to reduce energy consumption is also integrated into MAS-based smart grid simulators [19]. The abundance of work in this area implies the success of MAS in smart grid design simulation and modeling.

As far as the demand response issue is concerned, there are basically two types of solutions. One is based on the auction scheme, and the other is based on optimization [15, 16]. The auction scheme uses either a naive method or continuous double auction to allow the demands of loads to be satisfied by generators. The optimization method incorporates either conventional computational intelligence methods or novel methods. Conventional methods include simulated annealing (SA), genetic algorithm (GA), Tabu search (TS), ant colony optimization (ACO), and particle swarm optimization (PSO) [22]. Novel methods include model-predictive control (MPC) optimization [23, 24], thermal load modeling [25], autonomous distributed optimization [26], bilevel programming [27], load curtailment [28], neural networks [29], and hardware-in-the-loop simulation [30].

Researches on energy storage systems have been limited to the extension of their lifetime [31], battery mode control [32], and their usage in emergency operation of microgrid [33]. The use of energy storage systems in microgrid has also been investigated purely from the aspect of satisfying grid objectives [15, 16], without paying attention to the objectives of the energy storage systems. There is thus a lack of a complete infrastructure in which energy storage systems are considered in microgrids such that both the grid objectives and the storage goals are considered at the same time.

From the above survey on smart grid design, we can see that the main architecture is still a tightly integrated one, which lacks flexibility and scalability. In this work, we propose smart grid as a service (SGaaS), which leverages the service-oriented architecture of systems and makes it more flexible and scalable.

## 3. Smart Grid as a Service

Conventional electricity distribution power grids are gradually being transformed into smart grids. Smart grids introduce distributed energy resources (DER) and power users can decide how, when, and from where to acquire electricity. The utility service (centralized generation of electricity) is no longer the only available option for acquiring electricity. The main benefits of smart grids include reduction in overall cost of electricity (due to real-time awareness of electricity usage through advanced metering infrastructure (AMI)), reduction in global pollution (due to green renewable energy resources), higher reliability in electrical energy distribution or lesser probability of outages (due to smarter advanced distribution automation system (ADAS)), higher efficiency in energy distribution (due to shorter distribution paths and localized microgrids), and reduction in wasted electricity (due to peak leveling of utility power generation brought about by sharing of demands by DER).

Though smart grids present several benefits for both the utility and the power users, there are still several issues that need further research and design. Some typical issues include the restructuring of the power distribution grid to account for increasing renewable energy resources, the demand-response problem at microgrid, the smart grid levels, the quality of service (QoS) guarantee for all kinds of functional requirements such as load shedding [19] and load scheduling, and the flexibility and scalability of interconnecting users with the grid. Several methods have been proposed [15–19] for resolving the above issues including multiagent system- (MAS-) based architecture design and integrated design with centralized control. A novel design being proposed recently is the service-oriented architecture (SOA) for smart grids. In the rest of this section, we delve on how software engineering techniques can be used for developing the SOA for smart grids.

**Figure 2:**
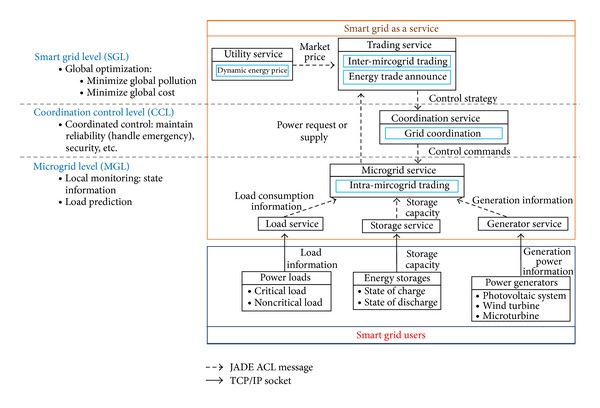
Smart grid as a service architecture.

The paradigm being proposed in this work is a smart grid as a service (SGaaS) which differentiates itself from state-of-the-art design in the scalability and reliability of the architecture. The architecture for SGaaS is shown in Figure 2, where the service-oriented architecture is divided into three levels corresponding to a contemporary smart grid design, including microgrid level (MGL), coordination control level (CCL), and smart grid level (SGL). In the following, we will describe the services that belong to each level. We will also discuss on how service-level agreements (SLA) can be achieved through agent-based negotiations and how service-level compositions (SLC) can be performed automatically through well-defined interfaces and agent-based automation.

### 3.1. Microgrid Level

The microgrid level is the lowest level in the hierarchy of the SOA of a smart grid and end users basically subscribe to the services at this level. The end users include power loads, energy storages, and power generators. There are four types of services at this level, namely, load service, storage service, generator service, and microgrid service, as described in the following. Note that service-level agreements and service-level compositions will be covered in Sections 4.1 and 4.2, respectively.

*(a) Load Service*. Power loads such as a normal residential home, a commercial site, or an industrial plant or a part thereof can subscribe to this service and as a result can make request for buying electrical power. The rate at which power is bought and the amount of power that can be bought will be either negotiated statically during SLA negotiations or determined dynamically during power trading. 

*(b) Storage Service*. Energy storage systems such as conventional batteries or batteries from plug-in electric vehicles (PEV) can subscribe to this service and as a result can make requests for buying or selling electrical power. The rate and amount of power sold/bought are also determined either statically during SLA negotiations or dynamically during power trading. 

*(c) Generator Service*. Power generators such as photovoltaic (PV) solar energy generation systems, wind turbines (WT), fuel cells (FC), and microturbines (MT) can subscribe to this service and as a result can sell electrical power. The rate and amount are also determined statically during SLA negotiations or dynamically during power trading. 

*(d) Microgrid Service*. This is not an end user service, and it cannot be subscribed by end users. It is subscribed by the three services described above, namely, load, storage, and generator services. A microgrid service is responsible for intra-microgrid trading, which means electricity trading among the load, storage, and generator services. The trading scheme or model is negotiated during SLA between the microgrid service and the participating services.

### 3.2. Coordination Control Level

This is a middle level that is responsible for maintaining grid-level constraints such as the voltage restrictions and substation requirements. There is only a single service in this level, namely, coordination service. There are basically two functionalities of this service as described in the following.

- This service checks the feasibility of each and every trading action determined by the upper smart grid level services before the actions are implemented by the microgrid level services. If constraints are violated by any trading action, this service will not allow the action to be implemented and will inform the upper smart grid level services to either redo the trading or declare no trading possible for the current time slot.
- This service is also responsible for microgrid isolation. It either directs a microgrid into island mode or helps a microgrid recover from island mode. The former is called intentional islanding and the latter is unintentional islanding. Island mode management could itself be an independent service; however, in this work, we prefer to include it into the coordination service for both simplicity, as well as grid reliability.

### 3.3. Smart Grid Level

This is the topmost level and is responsible for smart grid level inter-microgrid trading. There are two services, namely, utility service and trading service, which are described as follows. 

*(a) Utility Service*. This service represents the utility company, which can supply electricity power without any interruption, but at a much higher price compared to that of the electricity power from microgrids. The utility service declares its electricity price dynamically, which can be as short as once per hour or as long as once per day or per week. Contract-based power supply can also be negotiated between the trading service and the utility service. 

*(b) Trading Service*. This service is responsible for matching the power demands with the power supplies across microgrids. The power supplied by a microgrid can be directed to one or more microgrids that demand power. The power demand from a microgrid can be met by one or more microgrids that have excess power to supply. A microgrid service can subscribe to a trading service in a smart grid; however, it is required that the microgrid service also subscribe to the coordination service associated with that trading service. This requirement ensures that all microgrid services subscribing to the trading service abide by the coordination rules set by the said smart grid. The trading method can be either one of the following three, as typically found in contemporary system.

1. Matching: a simple matching between power demand and supply is performed. Electricity prices are fixed and determined at the time a microgrid joins the smart grid by subscribing to the trading service.
2. Bidding: a bidding (auction) is performed between the electricity sellers and buyers by the trading service. The auction result can be either the first price or the second price. The bids can be either hidden (sealed) or broadcast. Who sells first can be decided through contract-based priority settings among electricity sellers, which can be either storage services or generator services. Another alternative is a round-robin based scheduling of electricity sellers.
3. Optimization: a goal-oriented approach can be adopted by the trading service [15, 16]. The goals could include minimizing the overall electricity cost over time, the global estimated pollution due to the use of utility electricity, and/or the grid efficiency in distributing power. Well-known optimization methods that can be applied to smart grid optimization include simulated annealing (SA), genetic algorithm (GA), Tabu search (TS), ant colony optimization (ACO), and particle swarm optimization (PSO) [22].

## 4. Service Design Issues

The service-oriented architecture of smart grid as described in Section 3 still faces several typical problems. We focus on two main problems, namely, service-level agreements and service composition.

### 4.1. Service-Level Agreements

Service-level agreements (SLA) must be made when an end user subscribes to a service in the microgrid level. Some typical examples are as described as follows. 

*(a) Load SLA*. A load service needs to monitor and predict power demands of a power load. The SLA needs to include a guarantee by the service on the amount of electricity that can be supplied to the load. The rate at which monitoring is to be performed, the meantime between failure (MTBF) in electricity supply, and the time duration in which electricity supply is available are some of the terms that can be negotiated for SLA. The price at which electricity can be supplied is optional in the SLA because dynamic pricing can be negotiated during trading, either within the microgrid or across microgrids. Load control can also be negotiated in SLA, including load shedding and load scheduling, which will help reduce the overall cost of electricity for both the smart grid and the individual power loads. 

*(b) Storage SLA*. A storage service needs to monitor, predict, and schedule energy storage systems (ESS). The state of charge (SOC) of an ESS indicates the amount of electricity available in the ESS. SOC needs to be calibrated and monitored. Besides monitoring, storage service is also responsible for charging and discharging the ESS based on the predicted generation of power and the predicted power consumption of loads. 

*(c) Generator SLA*. A generator service needs to monitor and predict the amount of power generated by generators. Some generators can also be controlled, for example, fuel cells. The SLA could include a dynamic pricing scheme for the power generated by the generator, a priority setting for selling power, and the targeted buyers. A generator may choose whom to sell electricity. Such a choice is mostly due to the geographical restrictions and the interconnection infrastructure (which buyers are connected to which sellers).

Further, SLA is also required at the other levels. The trading-related SLA is as follows. 

*(d) Microgrid SLA*. The microgrid service needs to negotiate the intra-microgrid trading scheme with the participating services (load, storage, and generator). Quality-of-service (QoS) guarantees can be promised to the participating services in SLA. For example, the maximum latency between demand (request for electricity) and response (electricity supply) could be negotiated in SLA. 

*(e) Trading SLA*. The trading service negotiates the inter-microgrid trading scheme with the participating microgrid services. Provision of QoS guarantees can be made in SLA in accordance with the type of demands and responses.

### 4.2. Service Composition

The service-oriented architecture of a smart grid allows a user to choose and compose services as required. A smart grid user could be an individual end user (load/storage/generator) or a group of end users, along with microgrid and smart grid level service selections. For example, a user can choose to subscribe to one or more load/storage/generator services, along with a microgrid service, coordination service, and trading service. SLA for each service can be negotiated during service composition.

The major issues encountered during service composition include the following. 

*(a) Interface Discrepancy.* Due to the format variations (WSDL, SOAP, UDDI,…, etc.) in the requests made by power loads, energy storages, and power generators, composing services at the microgrid level require standardizing the interface between data producers and data consumers. A typical interface implemented by the authors in the microgrid system developed in cooperation with the government of Taiwan is as follows.

A text file is used for recording the power data for each week. Each day is divided into 96 time slots of 15 minutes each. The file consists of 7 rows (one for each day), and each row consists of 98 values. The first value is the calendar date, the second value is the name of the data source, and the rest 96 values are the power data for the 96 time slots of that day. 

*Example*. “20140608 LOAD23 15 20 16 19 ….” represents the power consumed by LOAD23 on 2014/6/8. The power data is in KWh/15 minutes. 

*(b) Conglomeration Issues*. Standardizing service interface as described above works only if the outputs of a service are a superset of the inputs of another service. For example, the power demand request made by a load service is a subset of the power data collected by a microgrid. When the inputs of a service *S* do not match the outputs of one of more services that *S* is dependent on, then some composition issues arise. Since there could be more than two services whose outputs need to be transformed into the inputs of a service, such a compound composition is called* conglomeration *in this work.

A typical example of conglomeration issues lies in the fact that an input of a service needs to be calculated from the outputs of one or more services. For example, in our smart grid design experience, the power generation service for photovoltaic (PV) systems needs the sunlight irradiance in w/m^2^; however, climate prediction services can only provide the global radiation in MJ/m^2^ and the duration of insolation in hours. We need to calculate the irradiance using the following formula, while taking care that whenever the irradiance is more than 1000 w/m^2^, then only 1000 w/m^2^ is considered since the solar panel cannot take more than that amount of irradiance:

(1) Irradiance(w/m2)  =Global  Radiation(MJ/m2)×106  Duration  of  Insolation(hr)×3600(sec/hr).

*(c) Location-Based Constraint Violation.* Due to the location of a service requestor and a service provider, the composition of the two services could violate location-based constraints. For example, a generator service and a load service cannot be composed if the power generated by the generator service cannot be transmitted to the power load subscribing to the load service. Such restrictions exist because the power grid is not fully connected; that is, not all generators are connected to all loads. Physical proximity is a major location constraint due to the inefficiencies involved in long distance transmission of power over grid lines. 

*(d) End-to-End Quality of Service Guarantee Violation*. Due to dynamic electricity pricing, real-time monitoring and control becomes a requisite of smart grids. However, currently, the information from smart meters is rarely real-time, most of the power consumption/storage/generation data are uploaded to cloud computing servers, and microgrid services access these data through web services, which are themselves prone to unacceptable delays. For example, a web service can be as long as 2 minutes or more if there is web proxy problems or security-related issues. New solutions are emerging in the form of constrained application protocol (CoAP) that allow wireless sensor nodes to communicate directly with each other, without going through the heavy-weight HTTP protocol stack. In the future, smart meters might be able to leverage on such light-weight protocols and real-time data information can be collected from the smart meters themselves, instead of from the cloud servers.

## 5. Implementation of Smart Grid as a Service

**Figure 3:**
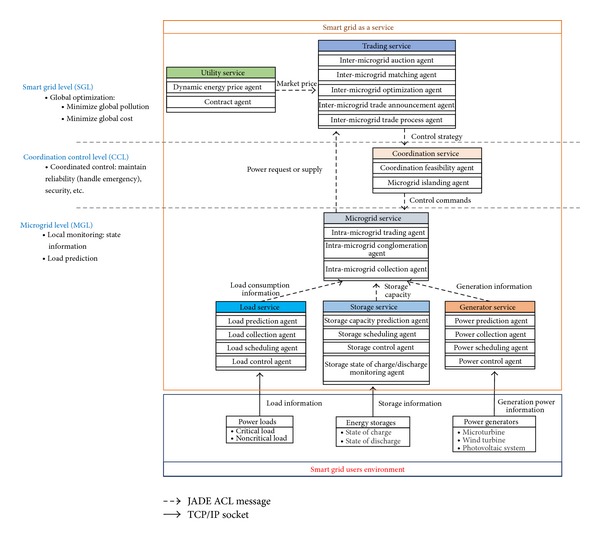
MAS-based smart grid service-oriented architecture.

The proposed smart grid as a service was designed as an SOA and implemented using multiagent system (MAS). In this work, the Java Agent Development Framework (JADE) [34] was used to develop our proposed architecture. JADE is a FIPA-compliant software framework that has the ability to realize distributed MAS. The MAS-based implementation of the service-oriented architecture for SGaaS is as shown in Figure 3.

The MAS-based SGaaS architecture consists of 3 levels, and each level contains one or more services that are composed of several agents. The agents deal with different events such as scheduling the residential power usage and monitoring the state of charge (SOC) of the storage devices. At the microgrid level, there are four types of services, namely, load service, storage service, generator service, and microgrid service. At the coordination control level, there is a single coordination service. At the smart grid level, there are two types of services, namely, utility service and trading service. The services at each level are described in detail in the following.

### 5.1. Microgrid Level (MGL)

*(1) Load Service.* Power loads are served through load services. Each power load such as an industrial plant, a part of a smart home, or a commercial unit must subscribe to a load service to meet its electrical power requirements. A load service is composed of four agents, including load prediction, load collection, load scheduling, and load control agents. To serve a power load, the service needs to predict the amount of power the load requires in a particular time slot. However, prediction is often based on history of power usage; thus, the service needs to collect historical power usage information from the load. To realize effective reduction in or scheduling of power usage, the service needs to be able to schedule and control loads. Load SLA is determined via negotiation a priori to load service subscription. The above-mentioned four functions of a load service are realized using the following four agents.

- *Load Prediction Agent.* This agent is responsible for predicting the future power requirements of loads, which are then provided to the microgrid service for requesting electricity. The agent predicts power requirements based on a linear regression analysis of historical power usage information and other environmental or contextual information such as temperature, day of the week, season, and geographical location [35].
- *Load Collection Agent.* The historical power usage information for a load is collected by this agent. The rates at which information is collected and the amount of information collected are negotiable in the corresponding load SLA. For example, in our current implementation, the rate is a multiple of 15 minutes and the information for a load is the amount of power used in a time slot. The dynamic pricing of electricity assumes a specific rate, say hour, half-hour, which can be used for load power usage information collection.
- *Load Scheduling Agent.* This agent is responsible for load scheduling and load shedding. The main goal is to mitigate the peak loads in a microgrid via load scheduling and/or shedding. The scheduling can be based on integer-linear programming, genetic algorithm, simulated annealing, or some other heuristics.
- *Load Control Agent.* This agent is responsible for electrical device control, including turning on/off the device and changing the execution mode of the device (such as increasing/decreasing the temperature of an air conditioner).

*(2) Storage Service*. This service is responsible for monitoring, scheduling, controlling, and predicting the capacity of energy storage systems. The service is composed of four agents whose functions are as follows.

- *Storage State of Charge/Discharge Monitoring Agent.* This agent is responsible for monitoring the state of charge/discharge of an ESS, so as to prevent over charge/discharge of batteries. Overcharge/discharge may result in damage of cells and degradation in recharge capabilities.
- *Storage Scheduling Agent. *This agent is responsible for scheduling the battery usage so as to reduce the overall cost of electricity use and also for increasing the lifetime of batteries. Normally, a tradeoff between the two must be achieved.
- *Storage Control Agent*. This agent is responsible for controlling and managing batteries; thus, it can govern the state of charge/discharge of all ESS in a microgrid.
- *Storage Capacity Prediction Agent.* This agent is responsible for predicting the battery capacities such that the microgrid service is aware of the amount of available power in batteries that can be used for meeting load requirements and/or the amount of power that must be purchased for storing into the batteries such that they can be used later during peak electricity price.

*(3) Generator Service.* Power generators such as PV, WT, FC, and MT are serviced by the generator service, which includes collection of power generation information, prediction of the amount of power to be generated in future time slots, and the scheduling and control of power generators. Four agents are used to realize the service as described in the following.

- *Power Collection Agent.* This agent is responsible for collecting and recording the power generation information such as the amount of power generated every time slot, where a time slot is similar to the time slot for power load information collection. The power generation information is used as historical data to predict future power generation.
- *Power Prediction Agent.* This agent is responsible for predicting the future power generation by the available sources, which is then provided to the microgrid service for the trading service. Power prediction can be performed based on simulations such as the MATLAB power simulators.
- *Power Scheduling Agent.* This agent is responsible for adjusting and scheduling the amount of power generation. Scheduling could be heuristic-based or optimization-based such as simulated annealing or genetic algorithm. The price and amount at which power is sold are negotiated during the subscription of the service.
- *Power Control Agent.* This agent is responsible for controlling power sources, if they are controllable. The purpose is to increase or decrease the amount of power generated. A typical example is the fuel cell power generators.

*(4) Microgrid Service*. A microgrid service is responsible for intra-microgrid trading upon receiving power buying requests from load/storage services and power selling requests from generator/storage services. The microgrid service is composed of three agents as described in the following.

- *Intra-Microgrid Collection Agent.* This agent is responsible for collecting all power load, power generation, and storage information within a microgrid. Interface discrepancy issue must be resolved, if any, by this agent. A cloud database is used to store all the information for further analysis by the intra-microgrid trading agent. Real-time collection and access of data are the main concerns that might affect the end-to-end QoS guarantee of this service.
- *Intra-Microgrid Trading Agent*. This agent is responsible for performing the power trading within a microgrid among the loads, generators, and storages. The trading mechanism can be either a simple matching between the buyers and sellers or a more complex auction scheme. This is decided during negotiation between the microgrid service and the participating services and recorded in the micro-grid SLA. After trading, the resulting power deficit or surplus in a microgrid is then submitted as a power buying request or a power selling request to the trading service at the upper smart-grid level.
- *Intra-Microgrid Conglomeration Agent*. This agent is responsible for the conglomeration of data from two or more other services. For example, the data from two sensor services can be merged into the data for a load prediction or generator prediction agent in corresponding services.

### 5.2. Coordination Control Level (CCL)

At the middle coordination control level of our proposed architecture, the single coordination service is responsible for maintaining grid-level constraints such as voltage restrictions and substation requirements such as location constraint. The service includes two agents.

*(1) Coordination Feasibility Agent*. This agent is responsible for ensuring the feasibility of trading actions determined by the upper smart grid level. The main goal is to guarantee the power quality in micro grids. Voltage restrictions and location constraints are some feasibility checks performed by this agent.

*(2) Microgrid Islanding Agent*. This agent is responsible for micro-grid isolation and recovery from isolation. It either directs a micro-grid into island mode or helps a micro-grid recover from island mode.

### 5.3. Smart Grid Level (SGL)

At the topmost smart grid level, there are two services, namely, utility service and trading service. The utility service consists of two agents, while the trading service consists of five agents, which are all described as follows.

*(1) Utility Service*

- Dynamic energy price agent: this agent is responsible for announcing the dynamic energy price from utility company once per scheduled time interval, for example, once per hour.
- Contract agent: this agent is responsible for negotiating a contract between the smart grid and the utility company. For example, a certain fixed amount of power can be purchased at a lower price; however, once the amount is exceeded the price will be much higher than the lower contract price.

*(2) Trading Service*

- Inter-microgrid trade announcement agent: this agent sends the start auction notification to each microgrid periodically. The period of notification depends on the period of price announcement by the dynamic energy price agent.
- Inter-microgrid trade process agent: this agent is responsible for negotiating and handling the trading process. One of the supported trading mechanisms can be chosen during negotiation, including auction, matching, and optimization, each of which is performed by an agent as described in the following.
- Inter-microgrid auction agent: this agent handles auction registration for all participating microgrids that need to buy or sell electrical power. The agent performs auction between participating microgrids using a first-price or a second-price auction method.
- Inter-microgrid matching agent: this agent uses a simple matching algorithm to satisfy the power demand-response requirements of microgrids. A buying microgrid can purchase electrical power from one or more selling microgrids. A selling microgrid can sell power to one or more buying microgrids. The match can be performed in a round-robin manner, based on dynamic or static priorities, or fairness-aware method.
- Inter-microgrid optimization agent: this agent uses an optimization algorithm to satisfy the demand-response requirements of microgrids. Some well-known optimization agents include SA, GA, TS, ACO, and PSO as mentioned in Section 3.

## 6. Conclusions

Conventional electricity power grid is static and not intelligent enough because it does not know how much and when power is required by users. The amount of power generated is much higher than the average users required. Thus, the power grid has to deal with the worst case of peak power usage, which results in not only a waste of electrical power, but also increases in environment pollution. As complementary efforts, smart grid tries to provide a more intelligent and efficient matching between the power generation and power demands for loads; nevertheless, the status quo of smart grid design is not yet fully mature because of the lack of a consummate infrastructure for communication and adaptation.

Accordingly, we proposed a novel smart grid as a service (SGaaS) which leverages the service-oriented architecture (SOA) of systems. SGaaS allows greater flexibility in system design and development. It also makes a system design more reliable due to the ease in which functions can be recovered via either resubscription of new services or recovery of failed services. The principal contributions of this work are to address two important issues of SOA, namely, service-level agreements and services composition. We demonstrated how service-level agreements can be achieved through agent-based negotiations and how service composition can be performed automatically via well-defined interfaces and agent-based automation. Further, we have shown how SGaaS can be realized using the state-of-the-art multiagent technology. We adopted the FIPA-compliant JADE multiagent system to implement our approach. Future work will consist of applying such a design to actual prototypes of smart grid.

## Figures and Tables

**Table 1:** Comparison of SOA in smart grids.

Literature	Hierarchical architecture			Trading process	System design feature	Simulator
	SGL	CCL	MGL			
Verschueren et al. [2]	Yes	Yes	Yes	N/A	Control strategies for power grid	OMNeT++

Chen et al. [3]	Yes	No	Yes	N/A	Generic service interfacing method for service standardization	None

Pham et al. [4]	Yes	No	Yes	N/A	Decision algorithm for power system	MATLAB and AREVA OTS

Enose [5]	Yes	Yes	Yes	N/A	Unified management system for smart grid	None

SGaaS (This work)	Yes	Yes	Yes	Auction, matching, or optimization	Distributed multiagent system	MATLAB and JADE

SGL: smart grid level, CCL: coordination control level, MGL: microgrid level, and N/A: not addressed.
